# Dietary total antioxidant capacity is associated with lower disease severity and inflammatory and oxidative stress biomarkers in patients with knee osteoarthritis

**DOI:** 10.1186/s41043-023-00450-x

**Published:** 2023-09-28

**Authors:** Farshad Amirkhizi, Soudabeh Hamedi-Shahraki, Mehran Rahimlou

**Affiliations:** 1 Department of Nutrition, Faculty of Public Health, Zabol University of Medical Sciences, Zabol, Iran; 2 Department of Epidemiology and Biostatistics, Faculty of Public Health, Zabol University of Medical Sciences, Zabol, Iran; 3 Department of Nutrition, Faculty of Medicine, Zanjan University of Medical Sciences, Zanjan, Iran

**Keywords:** Osteoarthritis, Antioxidant capacity, Inflammation, Food-frequency questionnaire, Oxidative stress

## Abstract

**Background:**

This study was designed to evaluate the association between dietary total antioxidant capacity and clinical and biochemical variables in patients with osteoarthritis.

**Methods:**

This cross-sectional study was conducted among 160 patients with mild-to-moderate knee osteoarthritis. The Likert version of the Western Ontario and McMaster Universities Osteoarthritis Index (WOMAC Index) was used to assess the severity of clinical symptoms in patients with knee osteoarthritis. The secondary outcomes included inflammatory and oxidative stress biomarkers. The participants' usual diets were assessed using a food frequency questionnaire (FFQ), and the dietary total antioxidant capacity (TAC) was calculated based on the ferric reducing antioxidant power method. Additionally, clinical and biochemical variables were evaluated using standard methods.

**Results:**

The mean age of the participants was 57.2 ± 8.1 years, and 55.6% of them were females. The dietary TAC scores in this study ranged from 3.67 to 24.72, with a mean of 12.05 ± 5.3. We found a significant inverse trend between the dietary TAC score and the total Western Ontario and McMaster Universities Osteoarthritis (WOMAC) score (*P* = 0.001), as well as the WOMAC stiffness (*P* = 0.008) and WOMAC physical function scores (*P* = 0.001). Furthermore, dietary TAC was inversely associated with serum concentrations of interleukin-6 (IL-6) (*β* = − 0.18, *P* = 0.020), tumor necrosis factor-α (TNF-α) (*β* = − 0.67, *P* < 0.001), matrix metalloproteinase-1 (MMP-1) (*β* = − 0.33, *P* < 0.001), and nuclear factor kappa-light-chain-enhancer of activated B cells (NF-κB) (*β* = − 0.22, *P* = 0.005) levels.

**Conclusion:**

The results of this study demonstrate an inverse association between dietary total antioxidant capacity and clinical and biochemical variables in patients with osteoarthritis.

## Background

Knee osteoarthritis (OA) is a prevalent chronic degenerative joint disorder characterized by progressive cartilage degradation, joint stiffness, and pain [1, 2]. It poses a significant burden on individuals' quality of life and imposes substantial health-care costs on society as a whole [3]. The prevalence of this disease is high in societies where the number of elderly and obese people is high [4]. According to the latest disability statistics report, hip and knee OA is one of the most important causes of disability among the adults [5–7].

The etiology of knee OA is multifactorial, involving genetic [8], mechanical, metabolic, and biochemical factors [9]. Among the various molecular mechanisms implicated in knee OA pathogenesis, oxidative stress has gained considerable attention [10, 11]. Additionally, some research showed that the expression of human leukocyte antigen [12], vitamin D receptor [13], insulin-like growth factor I [14], and cartilage oligomeric proteins can influence genetics to modify the susceptibility to OA [15].

Oxidative stress, an imbalance between the generation of reactive oxygen species (ROS) and the body's antioxidant defense mechanisms, is known to play a crucial role in the pathophysiology of knee OA [16, 17]. Persistent oxidative stress promotes the production of pro-inflammatory cytokines and chemokines, triggers the release of catabolic enzymes, and leads to the destruction of articular cartilage [18]. Consequently, strategies aimed at reducing oxidative stress, and inflammation may have therapeutic potential in managing knee OA progression [19].

Epidemiological studies have shown that dietary pattern is one of the important environmental factors in the occurrence of this disease, and the prevalence of knee OA is higher in societies that people adherence is from some dietary patterns such as western dietary patterns that contain high amounts of calories and saturated fatty acids and oxidant compounds [6, 20, 21]. Dietary antioxidants have emerged as a promising avenue for modulating oxidative stress and inflammation in various chronic diseases, including osteoarthritis [22]. The total antioxidant capacity (TAC) of the diet represents the cumulative effect of various antioxidants present in different foods and beverages [23]. High TAC diets have been associated with lower risk and improved management of several chronic conditions, such as cardiovascular disease [24], diabetes [25], and certain cancers [26]. However, the potential role of dietary TAC in knee OA remains relatively unexplored.

Understanding the association between dietary TAC and knee OA may have important clinical implications. Assessing the dietary TAC intake and its impact on disease severity, as well as inflammatory and oxidative stress biomarkers, in patients with knee OA could provide valuable insights into the role of nutrition in disease management and progression. Such investigations may help identify dietary strategies that complement existing therapeutic approaches, potentially improving patient outcomes, and reducing the need for more invasive interventions.

In this study, we hypothesized that higher dietary TAC can have positive effects in patients with knee OA. So, this study aimed to evaluate the association between dietary TAC with severity of disease and clinical biomarkers among the patients with knee OA.

## Methods and materials

### Participants

The participants comprised 160 males and females aged over 30 years with a diagnosis of mild-to-moderate bilateral primary knee osteoarthritis (OA). Knee OA was diagnosed based on the American College of Rheumatology (ACR) criteria [27] which include the presence of knee joint pain plus any three of the following six criteria: (1) age over 50 years; (2) presence of crepitus on active motion; (3) less than 30 min of morning stiffness; (4) bony tenderness; (5) bony overgrowth; and (6) no palpable warmth of synovium. For this cross-sectional study, patients were recruited from the hospitals or private clinics of Zabol University of Medical Sciences between January 2022 and June 2022. They were referred by rheumatologists based on the study's inclusion and exclusion criteria. The severity of knee OA was determined using the Kellgren–Lawrence (K–L) grading system (grades 0–4) [28]. According to this grading system, mild and moderate knee OA were considered as K–L grades 1–2 and 3, respectively.

### Sample size determination

To determine the sample size, we considered serum high-sensitivity C-reactive protein (hs-CRP) obtained from a study by Valtuena et al. [29], as a key-dependent variable. With a standard deviation (SD) of 2.1, effect size (d) of 0.3, and significance level (α) of 0.05, it was estimated that 160 subjects needed to be selected for the study.

### Inclusion and exclusion criteria

Inclusion criteria for the study participants were as follows: (1) willingness to participate in the study; (2) fulfillment of the ACR criteria for knee OA; (3) age greater than 30 years; and (4) experiencing knee pain for at least 3 months. Pregnant and lactating females, individuals with rheumatic diseases other than knee OA, and those taking antioxidant supplements such as selenium, carotenoids, and vitamins E and C within the 3 months prior to the study were excluded. Volunteers with a history of cardiovascular diseases, endocrine disorders (such as diabetes and hypo-/hyperthyroidism), cancer, and renal or liver dysfunction were also excluded. Participants following a specific diet, using fish oil supplements, or taking anti-inflammatory medications in the past 3 months were excluded as well. Additionally, individuals unable to comprehend and answer the questions or physically incapable of undergoing a physical examination were excluded from the study.

### Ethics approval

The study was conducted in compliance with the Declaration of Helsinki, and the research protocol was approved by the Ethics Committee of Zabol University of Medical Sciences (Ethics No.: IR.ZBMU.REC.1400.119). Prior to data collection, participants were informed about the study's aims and methodology and were asked to sign a written informed consent letter.

### Dietary assessment and calculation of dietary TAC

The usual diet information of patients was obtained through face-to-face interviews using a 168-item validated semi-quantitative food frequency questionnaire (FFQ) specifically designed based on commonly consumed Iranian foods. Detailed information about the design, food items, and validity of this questionnaire is described elsewhere [30, 31]. Briefly, the questionnaire recorded the amount and frequency of consumption of each food item during the preceding year on a daily, weekly, or monthly basis. Assistants helped patients estimate food quantities using calibrated household measurements (e.g., spoon, bowl, and ladles). The portion size of food items eaten by each patient was converted from household measures to grams. The intake of calories and nutrient content of foods was estimated using Nutritionist IV software (First Databank; Hearst, San Bruno, CA, USA) based on the modified US Department of Agriculture food composition for Iranian foods. Almost all foods in the participant list were coded, and for non-available foods, a similar item was coded.

In this study, dietary total antioxidant capacity (TAC) was calculated based on the ferric reducing antioxidant power (FRAP) method, which is an instrument for evaluating the capability of dietary antioxidants to reduce ferric to ferrous ions [32, 33]. The TAC values of foods were obtained from previously published papers that provided the antioxidant capacity for each food item, determined by FRAP. The FRAP values of foods are reported as millimoles per 100 g of each food item (mmol/100 g) [33]. For food items where TAC data were not directly available, we used the value of the nearest comparable food. Additionally, if any cooked food did not directly match a corresponding food in the database, the TAC value of a similar raw food was substituted. To calculate the dietary TAC for each patient, the consumption volume of each food item was multiplied by their related FRAP values and then summed up.

### WOMAC Index

The Likert version of the Western Ontario and McMaster Universities Osteoarthritis Index (WOMAC Index) was used to evaluate the severity of clinical symptoms in patients with knee OA [34]. The validity and reliability of the WOMAC Index in the Iranian population were determined [35]. The WOMAC Index is a disease-specific questionnaire that measures three dimensions: WOMAC pain (score range: 0–20) includes five questions regarding pain, WOMAC stiffness (score range: 0–8) includes two questions regarding stiffness, and WOMAC function (score range: 0–68) includes 17 questions that evaluate the degree of difficulty in performing daily activities. Each question is scored on an ordinal scale from zero to four, with higher scores indicating greater symptom severity or physical disability. The three subscales can be scored separately or as a total measure.

### Laboratory measurements

Fasting blood samples were taken from all study subjects after a 10- to 12-h fast and centrifuged at 3500 rpm (~ 2000 g) to separate the sera. Serum levels of interleukin 1-β (IL-1β), interleukin-6 (IL-6), and tumor necrosis factor-alpha (TNF-α) were assessed using human ELISA kits from DIA Source (Diaclone Inc., France), according to the manufacturer’s instructions. All intra-assay coefficients of variations (CVs) for serum interleukins and TNF-α were less than 7%. Serum high-sensitive C-reactive protein (hs-CRP) levels were evaluated based on the immunoturbidimetric method using the Pars Azmoon kit (Pars Azmoon Co., Tehran, Iran). Nuclear factor kappa-B (NF-kB) p65 was measured in peripheral blood mononuclear cell (PBMC) lysates using an ELISA kit from cell signaling (MA) according to the manufacturer’s protocol. Intra-assay CVs for serum hs-CRP and NF-kB were 6.8% and 9.2%, respectively.

Serum levels of matrix metalloproteinase-1 (MMP-1), matrix metalloproteinase-3 (MMP-3), and matrix metalloproteinase-13 (MMP-13) were measured using human ELISA kits (Boster Bio-sciences Co., Wuhan, China) [36, 37].

### Assessment of other variables

Data on general characteristics, including age, sex, smoking status, supplement use, duration of disease, and past medical history, were collected using a self-administered questionnaire. Bodyweight and height were assessed with minimal clothing and without shoes using the Seca scale (Germany) with an accuracy of 100 g and 0.5 cm, respectively. Body mass index (BMI) was calculated by dividing weight (in kilograms) by height squared (in square meters). To evaluate the physical activity levels of the participants, a short form of the International Physical Activity Questionnaire (IPAQ) was used [38].

### Statistical analyses

Patients were categorized into quartiles based on their overall dietary TAC score. The normality of the data distribution was checked using the Kolmogorov–Smirnov test and Q–Q plot. To compare general characteristics across quartiles of dietary TAC, one-way ANOVA was used for continuous variables, and the Chi-square test was used for categorical variables. Sex-, age-, and energy-adjusted means for dietary intakes across dietary TAC quartiles were compared using analysis of covariance (ANCOVA). To compare inflammatory markers and matrix metalloproteinases across dietary TAC quartiles, analysis of variance (ANOVA) was performed. ANCOVA with adjustment for age, sex, BMI, cigarette smoking, vitamin D and calcium supplement use, disease duration, physical activity, and energy intake was applied to compare clinical symptoms (total WOMAC score, WOMAC pain, WOMAC stiffness, and WOMAC physical function) across quartiles of dietary TAC. Multiple linear regression analyses in an adjusted model were used to explore the relationship between dietary TAC and inflammatory markers as well as matrix metalloproteinases. The adjusted model controlled for age (in years), sex (male/female), BMI (in kg/m2), disease duration (in years), energy intake (in kcal/d), vitamin D and calcium supplement use (yes/no), physical activity level (continuous), and cigarette smoking (smoker/nonsmoker) as confounders. All statistical analyses were performed using Statistical Package for the Social Sciences (SPSS Corp., version 18, Chicago, IL, USA), and P-values less than 0.05 were considered statistically significant.

## Results

### Subject's characteristics

In the present study, 160 patients with knee OA participated. The mean age of participants was 57.2 ± 8.1 years, and 55.6% of them were females. The dietary total antioxidant capacity (TAC) score in this study ranged from 3.67 to 24.72, with a mean and standard deviation of 12.05 ± 5.3. The dietary TAC in the first, second, third, and fourth quartiles was < 8.02, 8.02–11.39, 11.40–15.43, and ≥ 15.44, respectively. General characteristics of participants by quartiles of dietary TAC score are indicated in Table 1. There were no significant differences in the mean age, weight, body mass index (BMI), physical activity level, and disease duration across quartiles of dietary TAC. Additionally, differences in the distribution of current smokers, patients using vitamin D and calcium supplements, and those with obesity were non-significant when comparing different quartiles of dietary TAC.

**Table 1:** General characteristics of patients with knee osteoarthritis across quartiles of dietary TAC^a^

Variables	Quartiles of dietary TAC^b^				*P*^c^
	Q1 (*n* = 40)	Q2 (*n* = 40)	Q3 (*n* = 40)	Q4 (*n* = 40)	
Females, n (%)	22 (55.0)	24 (60.0)	19 (47.5)	24 (60.0)	0.638
Age (yr)	57.4 ± 9.6	57.9 ± 7.3	56.6 ± 8.4	57.1 ± 7.4	0.916
Weight (kg)	81.5 ± 13.6	82.0 ± 12.9	83.4 ± 11.8	81.3 ± 13.1	0.885
BMI (kg/m^2)^	27.7 ± 3.0	28.1 ± 3.1	27.6 ± 2.8	28.2 ± 3.3	0.725
Obesity, n (%)^d^	9 (72.5)	32 (80.0)	30 (75.0)	31 (77.5)	0.875
Vitamin D supplement use, n (%)	20 (50.0)	17 (42.5)	21 (52.5)	16 (40.0)	0.635
Calcium supplement use, n (%)	19 (47.5)	14 (35.0)	15 (37.5)	11 (27.5)	0.319
Current smoker, n (%)	9 (22.5)	7 (17.5)	9 ( 22.5)	8 (20.0)	0.936
Physical activity (MET-h/week)	29.0 ± 6.6	28.5 ± 6.8	30.1 ± 7.3	29.1 ± 6.2	0.760
Knee osteoarthritis duration (yr)	7.2 ± 4.8	6.7 ± 2.6	7.1 ± 2.7	6.0 ± 3.2	0.442

TAC, total antioxidant capacity and BMI, body mass index

^a^All values are expressed as means ± standard deviations unless indicated

^b^Quartile cut points of dietary TAC are as follows: first quartile, < 8.02; second quartile, 8.02–11.39; third quartile, 11.40–15.43; and fourth quartile, ≥ 15.44

^c^Resulted from one-way ANOVA for continuous variables and Chi-square test for categorical variable

^d^Obesity was defined as BMI ≥ 25 kg/m^2^

*P* < 0.05 was considered significant

### Dietary intakes

Dietary intakes of patients with knee OA across quartiles of dietary TAC are displayed in Table 2. Patients assigned to the highest category of dietary TAC had higher daily intake of energy (*P* < 0.001), carbohydrates (*P* = 0.001), vitamins C (*P* < 0.001) and A (*P* < 0.001), and refined grains (*P* = 0.002) compared to those in the lowest quartile. Furthermore, those in the highest quartile of dietary TAC consumed a higher amount of fruits (*P* < 0.001) and vegetables (*P* < 0.001) compared to patients in the lowest quartile. No other noticeable differences were found in dietary intakes across quartiles of dietary TAC.

**Table 2:** Dietary intakes of patients with knee osteoarthritis across quartiles of dietary TAC^a^

Variables	Quartiles of dietary TAC^b^				*P*^c^
	Q1 (*n* = 40)	Q2 (*n* = 40)	Q3 (*n* = 40)	Q4 (*n* = 40)	
Total energy intake (kcal/d)^d^	2038 ± 75	2429 ± 81	2371 ± 65	2584 ± 81	< 0.001
*Nutrients*					
Carbohydrate (g/d)	272 ± 4	308 ± 6	329 ± 6	339 ± 6	0.001
Protein (g/d)	71.5 ± 1.9	72.2 ± 2.1	70.7 ± 2.0	73.6 ± 2.3	0.453
Fat (g/d)	103.4 ± 3.6	96.5 ± 3.1	97.8 ± 3.4	100.5 ± 3.7	0.238
Vitamin C (mg/d)	89.3 ± 6.8	112.7 ± 12.2	148.5 ± 12.8	171.4 ± 15.2	< 0.001
Vitamin A (RAE/d)	326 ± 30	419 ± 33	581 ± 43	793 ± 58	< 0.001
Vitamin E (mg/d)	19.8 ± 1.1	20.4 ± 1.4	21.2 ± 1.4	18.4 ± 1.3	0.284
Zinc (mg/d)	7.1 ± 0.8	8.2 ± 1.0	7.8 ± 0.9	8.5 ± 0.8	0.142
Iron (mg/d)	11.8 ± 0.8	12.2 ± 0.9	13.2 ± 1.0	13.1 ± 1.1	0.286
Calcium (mg/d)	897 ± 34	976 ± 45	925 ± 53	1012 ± 55	0.327
*Food groups*					
Whole grains (g/d)	97 ± 10	126 ± 13	110 ± 13	117 ± 15	0.293
Refined grains (g/d)	318 ± 26	338 ± 30	408 ± 29	431 ± 32	0.002
Fruits (g/d)	181 ± 15	237 ± 16	279 ± 18	291 ± 20	< 0.001
Vegetables (g/d)	191 ± 14	216 ± 16	284 ± 21	311 ± 20	< 0.001
Legumes and nuts (g/d)	40.7 ± 3.1	37.5 ± 3.2	44.9 ± 3.4	41.7 ± 2.9	0.317
Meats (g/d)	81.6 ± 6.8	94.4 ± 8.3	90.5 ± 7.8	87.5 ± 6.4	0.246
Dairy products (g/d)	182 ± 7	190 ± 11	184 ± 9	187 ± 15	0.427
Dietary TAC (mmol/d)	5.5 ± 0.2	10.1 ± 0.2	13.4 ± 0.2	19.2 ± 0.4	

TAC, total antioxidant capacity; IL-1β, interleukin-1β; IL-6, interleukin-6; TNF-α, tumor necrosis factor-α; hs-CRP, high-sensitivity C-reactive protein; NF-κB p65, nuclear factor-kappa B (p65); MMP-1, matrix metalloproteinase-1; MMP-3, matrix metalloproteinase-3; and MMP-13, matrix metalloproteinase-13

^a^All values are expressed as means ± standard deviations

^b^Quartile cut points of dietary TAC are as follows: first quartile, < 8.02; second quartile, 8.02–11.39; third quartile, 11.40–15.43; and fourth quartile, ≥ 15.44

^c^Resulted from analysis of variance (ANOVA)

*P* < 0.05 was considered significant

### Association of dietary TAC with inflammatory markers and matrix metalloproteinases

Table 3 illustrates the means of serum matrix metalloproteinases and inflammatory markers of patients with knee OA across quartiles of dietary TAC. The mean serum concentrations of IL-6 (P-trend = 0.007), TNF-α (*P* < 0.001), as well as NF-κB concentrations in PBMCs lysates (*P* = 0.005) showed decreasing trends across the increase in dietary TAC quartile categories. Moreover, the serum concentrations of MMP-1 also exhibited decreasing trends across the increase in dietary TAC quartile categories (*P* = 0.001). No significant regular trends were found regarding IL-1β, hs-CRP, MMP-3, and MMP-13 across dietary TAC quartile categories.

**Table 3:** Serum levels of inflammatory markers and matrix metalloproteinases in patients with knee osteoarthritis across quartiles of dietary TAC^a^

Variables	Quartiles of dietary TAC^b^				*P*-trend^c^
	Q1 (*n* = 40)	Q2 (*n* = 40)	Q3 (*n* = 40)	Q4 (*n* = 40)	
IL-1β (pg/ml)	14.0 ± 7.5	12.8 ± 5.9	14.7 ± 8.4	13.3 ± 7.0	0.937
IL-6 (pg/ml)	10.8 ± 5.7	10.0 ± 5.7	9.4 ± 4.3	7.8 ± 3.9	0.007
TNF-α (pg/ml)	35.7 ± 6.4	27.5 ± 4.5	25.7 ± 4.4	22.8 ± 3.6	< 0.001
hs-CRP (mg/l)	5.52 ± 3.55	4.85 ± 2.74	4.91 ± 3.21	4.38 ± 2.51	0.119
NF-кB p65 (arbitrary unit)	1.94 ± 0.64	1.63 ± 0.46	1.71 ± 0.51	1.55 ± 0.54	0.005
MMP-1 (ng/ml)	7.69 ± 3.15	6.22 ± 3.26	6.13 ± 2.67	4.79 ± 2.53	0.001
MMP-3 (ng/ml)	13.6 ± 4.2	12.5 ± 3.1	13.6 ± 4.6	12.3 ± 2.9	0.258
MMP-13 (ng/ml)	0.88 ± 0.58	0.69 ± 0.25	0.80 ± 0.38	0.68 ± 0.30	0.101

TAC, total antioxidant capacity; IL-1β, interleukin-1β; IL-6, interleukin-6; TNF-α, tumor necrosis factor-α; hs-CRP, high-sensitivity C-reactive protein; NF-κB p65, nuclear factor-kappa B (p65); MMP-1, matrix metalloproteinase-1; MMP-3, matrix metalloproteinase-3; and MMP-13, matrix metalloproteinase-13

^a^All values are expressed as means ± standard deviations

^b^Quartile cut points of dietary TAC are as follows: first quartile, < 8.02; second quartile, 8.02–11.39; third quartile, 11.40–15.43; and fourth quartile, ≥ 15.44

^c^Resulted from analysis of variance (ANOVA)

*P* < 0.05 was considered significant

The results obtained from the multiple linear regression analyses confirm the aforementioned relationships (Table 4). Dietary TAC was inversely associated with serum concentrations of IL-6 (*β* = − 0.18, *P* = 0.020), TNF-α (*β* = − 0.67, *P* < 0.001), and MMP-1 (*β* = − 0.33, *P* < 0.001), as well as NF-κB concentrations in PBMCs lysates (*β* = − 0.22, *P* = 0.005), after adjustment for potential confounders.

**Table 4:** Results of multiple linear regression analyses that evaluated the association between dietary TAC with inflammatory markers and matrix metalloproteinases (*n* = 160)^a^

Variables	Dietary TAC		
	B (S.E.)	β	*P*^a^
IL-1β (pg/ml)	− 0.18 (0.5)	− 0.03	0.724
IL-6 (pg/ml)	− 0.83 (0.35)	− 0.18	0.020
TNF-α (pg/ml)	− 4.07 (0.36)	− 0.67	< 0.001
hs-CRP (mg/l)	− 0.36 (0.21)	− 0.13	0.093
NF-кB p65 (arbitrary unit)	− 0.11 (0.04)	− 0.22	0.005
MMP-1 (ng/ml)	− 0.91 (0.21)	− 0.33	< 0.001
MMP-3 (ng/ml)	− 0.41 (0.27)	− 0.12	0.139
MMP-13 (ng/ml)	− 0.05 (0.03)	− 0.12	0.124

TAC, total antioxidant capacity; IL-1β, interleukin-1β; IL-6, interleukin-6; TNF-α, tumor necrosis factor-α; hs-CRP, high-sensitivity C-reactive protein; NF-κB p65, nuclear factor-kappa B (p65); MMP-1, matrix metalloproteinase-1; MMP-3, matrix metalloproteinase-3; MMP-13, matrix metalloproteinase-13; B, unstandardized coefficient; and S.E., standard error

^a^Adjusted for age (continuous), sex (male/ female), BMI (kg/m^2^), disease duration (continuous), energy intake (kcal/d), vitamin D and calcium supplement use (yes/ no), physical activity level (continuous), and cigarette smoking (smoker/nonsmoker)

*P* < 0.05 was considered significant

### Association of dietary TAC with clinical symptoms

**Fig. 1:**
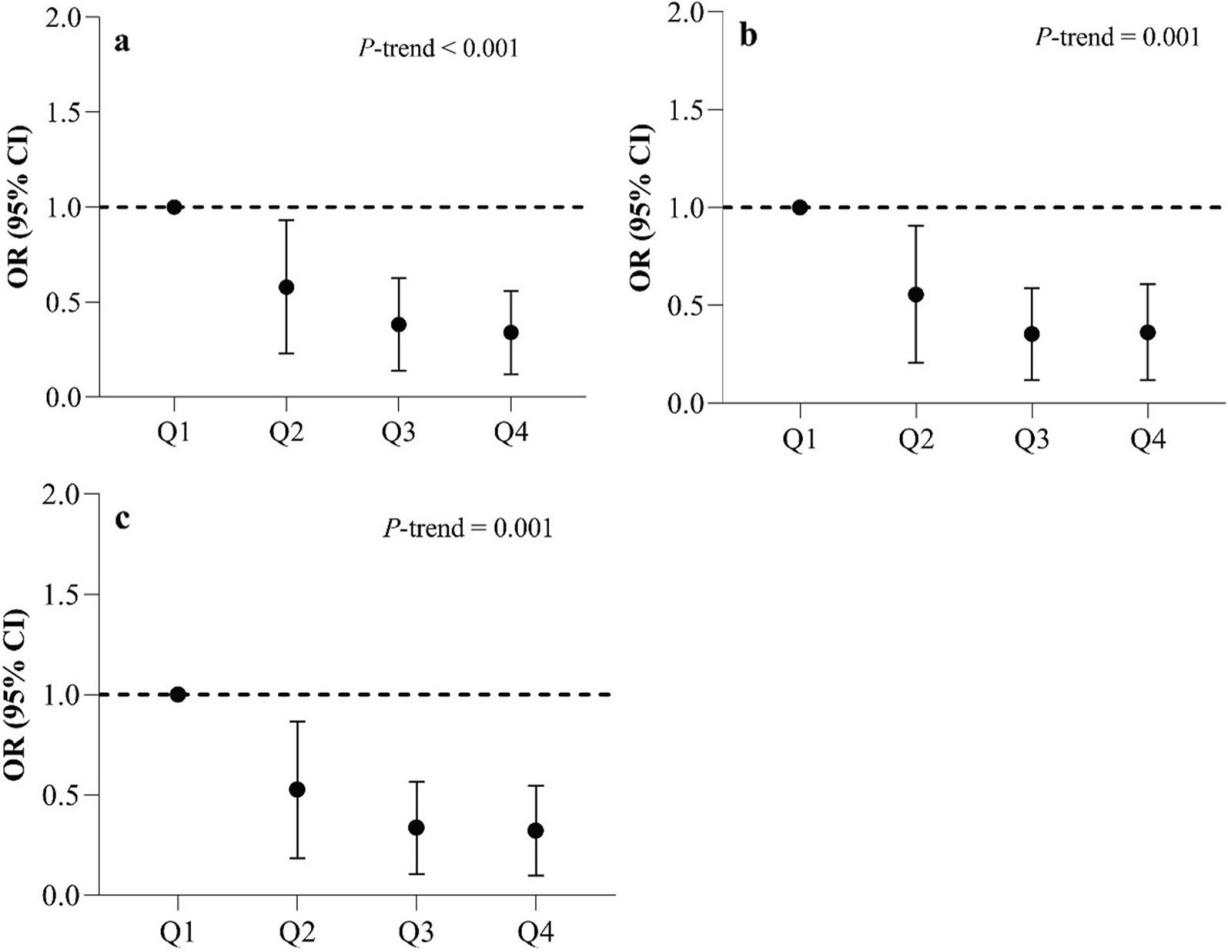
McMasters Universities (WOMAC) scores (total, pain, stiffness, and physical function) of patients with knee osteoarthritis across quartiles (Q) of dietary total antioxidant capacity. Data are shown as OR. The number of participants was 40 in each quartile. *P*-trend values were obtained from analysis of covariance (ANCOVA) with adjustment for age, sex, BMI, cigarette smoking, vitamin D and calcium supplement use, disease duration, physical activity, and energy intake. Quartile cut points of dietary TAC are as follows: first quartile, < 8.02; second quartile, 8.02–11.39; third quartile, 11.40–15.43; and fourth quartile, ≥ 15.44. *P* < 0.05 was considered significant

Figure 1 represents the scores of the Western Ontario and McMaster Universities Osteoarthritis Index (WOMAC) (total, pain, stiffness, and physical function) of patients with knee OA across quartiles of dietary TAC. After adjustment for potential confounding factors, a significant inverse regular trend was observed between dietary TAC score and the total WOMAC score (*P* = 0.001), as well as WOMAC stiffness (*P* = 0.008) and WOMAC physical function (*P* = 0.001). Nonetheless, no significant association was found between dietary TAC score and WOMAC pain score.

## Discussion

To our knowledge, this is the first study designed to evaluate the association between dietary TAC and disease symptoms as well as clinical biomarkers among patients with knee OA. The results of the present study showed that patients in the highest quartile of dietary TAC had lower concentrations of some inflammatory factors, including IL-6, TNF-α, MMP-1, as well as NF-κB. We also found a significant inverse association between dietary TAC and clinical symptoms among patients with knee OA.

As mentioned, oxidative stress is one of the main risk factors involved in the pathogenesis of chronic diseases such as OA. In OA, oxidative stress and ROS damage extracellular constituents, cellular membranes, and nucleic acids, impairing metabolic processes, altering the structure of proteins, and accumulating damaged proteins in the tissue [39]. Organisms that utilize oxygen for energy production have developed complex mechanisms for removing ROS. Enzymatic and non-enzymatic antioxidants, such as SOD, CAT, GPX, GSH, NQO1, PON, vitamin C, vitamin E, and carotenoids, play a role in these functions [16, 39]. In patients with OA, polymorphonuclear leukocytes are activated, generating excessive amounts of ROS. Increased levels of ROS can induce lipid peroxidation and cause damage to tissues [40].

As mentioned, we found that participants who adhered to a dietary pattern containing a higher number of antioxidants, such as selenium, vitamin C, vitamin A, and other antioxidants, were able to reduce serum levels of some inflammatory biomarkers. Several studies have shown that traditional dietary patterns characterized by high intake of fruits, vegetables, whole grains, and fish are associated with a lower risk of inflammatory diseases [41, 42]. Conversely, it has been reported that a western dietary pattern, which contains lower amounts of antioxidant nutrients, is associated with a higher risk of inflammatory diseases such as OA [43, 44]. In a longitudinal study, Xu et al. reported that adherence to western and prudent dietary patterns was significantly associated with radiographic and symptomatic progression of knee OA [21].

Our study showed that patients in the highest quartile of TAC had lower serum levels of inflammatory factors. Allkan et al. also found a significant inverse association between dietary TAC and some inflammatory markers among patients with cancer [45]. Interestingly, our results regarding the preventive properties of dietary TAC against inflammation-related damage were supported by other studies [46, 47]. It has also been shown in some studies that the serum levels of antioxidants in patients with arthritis are lower than in healthy individuals [48]. Additionally, diet is a key source of inflammation, and the dietary inflammatory index (DII) is a useful tool for assessing the overall inflammatory potential of an individual's diet. A study reported that higher DII scores, indicating a more pro-inflammatory diet, are associated with a higher incidence of frailty, particularly in men [49].

One of the main mechanisms involved in the protective effects of dietary antioxidants against OA is the prevention of 8-hydroxydeoxyguanosine production from DNA damage by inhibiting the production of ROS [50]. Antioxidant compounds in the diet, such as beta-carotene and alpha-tocopherol, have been shown in many studies to exert inhibitory effects on the activity and expression of nuclear transcription factor κB (NF-κB), which plays an important role in inducing the inflammatory process [51]. Vitamin E in high TAC diets can exert anti-inflammatory effects by inhibiting the arachidonic acid pathway, a precursor to the synthesis of inflammatory prostaglandins and leukotrienes [52]. Additionally, high TAC diets usually contain high amounts of soluble and insoluble fibers, which exert anti-inflammatory effects through fermentation in the colon environment and the production of short-chain fatty acids (SCFAs) with strong anti-inflammatory properties [53]. Positive changes in the gut microbiome following high-fiber diets have also been shown to have anti-inflammatory effects [54]. Oxidative stress through ROS activation of inflammatory cascades, including increasing the expression of nuclear factor-kB and cytokine production, has been demonstrated [55, 56]. We did not find any significant association between hs-CRP concentration and dietary TAC, which contradicts some previous studies [46, 57]. However, Porhan et al. reported in a case–control study among university male students that there was no significant association between dietary TAC and hs-CRP concentration [58].

We found a significant inverse association between TAC and MMP-1 concentration. Patients with OA experience excruciating pain as a result of extracellular matrix (ECM) degradation in synovial joints, particularly in the knee, hands, and hips. Immune and joint cells produce multiple inflammatory agents, including TNF-α and interleukins. These pro-inflammatory cytokines promote the synthesis of matrix metalloproteinases (MMPs), which are enzymes capable of breaking down all ECM constituents [40]. Collagenases such as MMP-1 and MMP-13 play predominant roles in OA because they are rate-limiting in the process of collagen degradation [59]. In line with our findings, Verma et al. reported that dietary antioxidants can suppress the growth of malignant cells by inhibiting the activity of MMPs [60].

Based on our knowledge, the present study was the first to evaluate the association between dietary TAC and clinical and biochemical variables among patients with knee OA. We selected newly diagnosed patients to reduce the likelihood of diet changes. Moreover, to minimize the effects of multiple confounding variables, we adjusted the results for several potential covariates. However, there were some limitations in our study. Firstly, since the nature of this study is cross-sectional, we cannot prove that the reported associations are causal, although we controlled for several potential covariates. Secondly, to assess dietary antioxidant intake, an FFQ has been used, which is dependent on the patient's memory and the interviewer's skill. Finally, other confounding factors that were not adjusted in the analysis, such as genetics and stress, may affect the accuracy of the results.

## Conclusion

This study highlights an important association between TAC and disease severity, as well as inflammatory and oxidative stress biomarkers, in patients with OA. The findings suggest that higher dietary TAC is associated with lower disease severity, reduced stiffness and physical dysfunction, and decreased levels of inflammatory and oxidative stress markers. Further studies specially prospective studies with a larger population are needed to confirm our findings.

## Data Availability

Data generated or analyzed during this study are included in this article and are available from the corresponding author on reasonable request.

## Abbreviations

ANCOVA: Analysis of covariance
ACR: American College of Rheumatology
BMI: Body mass index
ECM: Extracellular matrix's
IL-6: Interleukin-6
IPAQ: International Physical Activity Questionnaire
IL-1β: Interleukin 1-β
MMP-1: Matrix metalloproteinase-1
hs-CRP: High-sensitive C-reactive protein
GPX: Glutathione peroxidase
OA: Osteoarthritis
NF-κB: Nuclear factor kappa-light-chain-enhancer of activated B cells
NUO1: NADPH ubiquinone oxidoreductase
FRAP: Ferric reducing ability of plasma
FFQ: Food frequency questionnaire
ROS: Reactive oxygen species
TAC: Total antioxidants capacity
TNF-α: Tumor necrosis factor-alpha
WOMAC: Western Ontario and McMaster Universities Osteoarthritis
PBMCs: Peripheral blood mononuclear cells
